# Efficacy and safety of a fixed-dose papain and alpha-amylase combination in patients with functional dyspepsia: a real-world study

**DOI:** 10.3389/fgstr.2026.1954358

**Published:** 2026-10-28

**Authors:** S. Krishna Deepak, Ankur Jain, Sangitanjan Dutta, Abhinav Jain, Soham Kshirsagar, Rashi Joshi, Archana Toppo

**Affiliations:** 1 Biological E Limited, Hyderabad, India; 2 AJ Gastro and Liver Centre, Delhi, India; 3 Guwahati Medical College and Hospital (GMCH), Guwahati, India; 4 Gastro1 Hospital, Ahmedabad, India

**Keywords:** alpha-amylase, dyspepsia symptom relief, dyspepsia symptom score, fixed-dose combination, functional dyspepsia, papain

## Abstract

**Background:**

Functional dyspepsia (FD) is highly prevalent and often inadequately managed because conventional therapies may have limited efficacy or tolerability. Enzyme-based therapies offer a physiologically relevant alternative by supporting digestion. This study evaluated the real-world effectiveness and safety of a fixed-dose combination (FDC) of papain and alpha-amylase in patients with FD.

**Methodology:**

This prospective, multicenter, single-arm intervention study enrolled adults with FD (Rome IV criteria) across four Indian centers. Patients received the FDC for 12 days. The primary endpoint was a ≥50% reduction in composite symptom score. Secondary endpoints included symptom resolution, time to relief, safety, and subgroup outcomes. Assessments were conducted at baseline, Day 7, and Day 12.

**Results:**

Of 160 enrolled patients, 144 (90%) completed the study. Mean symptom scores declined significantly from 9.35 ± 1.42 (baseline) to 0.15 ± 0.35 (Day 12; *p*< 0.0001), indicating 98.4% improvement. Clinical response was achieved in all patients by Day 7 and sustained on Day 12, with 85.4% achieving excellent response. The arithmetic mean (unweighted) of domain-specific resolution percentages reached 98.7% by Day 12. Median time to initial relief was approximately 4 hours. Adverse events were mild and reported in 12.5% of patients.

**Conclusion:**

The FDC demonstrated short-term rapid, substantial, and consistent symptom relief with favorable tolerability in patients with FD.

## 1 Introduction

Functional dyspepsia (FD) is a chronic disorder of gastroduodenal function characterized by bothersome postprandial fullness, early satiation, epigastric pain, or epigastric burning in the absence of structural disease. It is diagnosed when one or more of these symptoms are present for at least 3 months, with symptom onset at least 6 months before diagnosis (1). Based on predominant symptoms, FD is classified into postprandial distress syndrome (PDS) and epigastric pain syndrome (EPS), although overlap between these subtypes is common (1, 2). The Rome IV criteria recognize early satiation, postprandial fullness, and EPS symptoms as bothersome and emphasize that FD classification includes PDS, EPS, and their overlap (3).

The global pooled prevalence of FD between 1990 and 2020 was estimated at 8.4% (95% CI, 7.4%–9.5%), with developing nations reporting a higher burden than developed nations (9.1% vs 8.0%). Regional variation was also notable, with prevalence figures ranging from 8% to 23% across Asia (4, 5). In 2023, India presented a significant burden of dyspepsia, with an estimated prevalence ranging from 7.6% to 49% (6). Community-based studies commonly report the prevalence of uninvestigated dyspepsia, as most affected individuals are likely to have FD in the absence of identifiable organic causes (7). A 2023 pan-India, multicenter, observational study spanning 29 states identified FD in 78.5% of 3739 patients (6). Dyspepsia prevalence in India was reported at 30.4% in 2001, rising to 49% in a multicenter community-based study conducted in 2008 (8, 9).

Current treatment strategies include proton pump inhibitors (PPIs), histamine type-2 receptor antagonists (H2RAs), prokinetic agents, and neuromodulators (10, 11). However, these approaches have significant limitations. PPIs demonstrate only marginal superiority over placebo (12). Prokinetics such as cisapride, domperidone, and itopride have been associated with adverse effects, including cardiac events, diarrhea, abdominal pain, constipation, or nausea, limiting their long-term use (11, 13). The limited efficacy, adverse effects, poor long-term tolerability, and refractoriness of many patients to conventional therapies call for alternative treatment approaches (12).

In recent years, enzyme-based therapies have emerged as a physiologically rational approach for the management of FD (14, 15). Papain, a proteolytic enzyme derived from *Carica papaya*, facilitates protein digestion and has demonstrated anti-inflammatory properties (16). Alpha-amylase enhances carbohydrate breakdown, potentially reducing postprandial symptoms related to delayed or incomplete digestion (17, 18). Previous literature suggests that digestive enzyme supplementation may improve dyspeptic symptoms, with fixed-dose combinations (FDCs) offering theoretical advantages over single-mechanism approaches by simultaneously involving multiple digestive pathways (14, 19). However, robust real-world data specifically evaluating this combination in Indian patients with FD remain limited. Given the high prevalence of FD in India, challenges associated with conventional therapies, and the potential benefits of enzyme supplementation, this study aimed to evaluate the clinical effectiveness and safety of an FDC of papain and alpha-amylase (Bestozyme^®^ syrup) in Indian healthcare settings.

## 2 Methods

### 2.1 Study design

This real-world, prospective, multicenter, single-arm interventional study enrolled 160 patients with FD across four study centers in India (Bengaluru, Delhi, Ahmedabad, and Guwahati). The study was conducted between 12 June and 04 December 2025 in accordance with Good Clinical Practice guidelines and the principles of the Declaration of Helsinki. The study protocol was reviewed and approved by independent ethics committees. All patients provided written informed consent before participation in the study.

### 2.2 Study population

Adult patients aged 18–70 years diagnosed with FD according to the Rome IV criteria were enrolled in the study (3, 20). The patients had a history of FD for 1–12 years. Pregnant or lactating women, patients who had participated in other clinical trials, and patients who used medications affecting gastrointestinal motility (except FD treatments) were excluded from the study. Patients with hypersensitivity to the study drug; those consuming alcohol or smoking; and patients with other gastrointestinal pathologies, including gastroesophageal reflux disease, irritable bowel syndrome, cardiovascular disease, chronic hepatic or pancreatic disease, autoimmune disorders, genetic metabolic diseases, or other significant medical conditions that could compromise patient safety or study validity were also excluded.

#### 2.2.1 Patient enrollment and follow-up

All patients underwent screening at baseline (Visit 1, Day 0), during which sociodemographic data (age and sex), clinical symptoms, disease duration, comorbidities, concomitant medications, and relevant medical history were recorded. Baseline symptom severity was assessed using an indigestion questionnaire (**Supplementary Table S1**). Follow-up assessments were conducted on Day 7 (Visit 2) and Day 12 ± 2 days (Visit 3). At each follow-up visit, the frequency and severity of FD symptoms were collected using the same questionnaire (**Supplementary Table S1**) to ensure consistency in outcome measurement. Any changes in treatment regimen, treatment compliance issues, and adverse events (AEs) were also documented throughout the study.

#### 2.2.2 Intervention

All patients received 10 mL of the FDC of papain and alpha-amylase (Bestozyme^®^ syrup) orally twice daily with meals for 12 days. Each 10-mL dose contained 150 mg of alpha-amylase (fungal diastase 1:800), corresponding to the ability to digest approximately 120 g of cooked starch. Fungal diastase also possesses ancillary proteolytic and lipolytic activities. Each 10-mL dose additionally contained 120 mg of papain, providing approximately 720, 000 papain units based on a minimum activity of 6000 units/mg. The administered dose was within the recommended maximum of 2, 400, 000 papain units per single dose and 7, 200, 000 papain units per day.

### 2.3 Study endpoints

The primary endpoint was treatment effectiveness, defined as the proportion of patients demonstrating clinically significant improvement in FD symptoms, as assessed by a ≥50% reduction in the composite symptom score from baseline to Day 12 (21, 22). The symptom-based questionnaire used in this study was a study-specific instrument, and the composite symptom score was constituted based on all 11 symptoms assessed in this questionnaire. Each of the 11 symptoms displayed in the questionnaire (bloating, nausea/vomiting, regurgitation, heartburn, burping, stomach pain, postprandial fullness/early satiety, gas/flatulence, feeling of stress, constipation, and sleep/overall health quality; **Supplementary Table S1**) was recorded dichotomously as present (Yes; score = 1) or absent (No; score = 0) at baseline, Day 7, and Day 12. The composite symptom score, which reflects the number of symptoms absent or present at a given visit regardless of baseline status, was calculated as the unweighted mean of the 11 item scores, yielding a possible range of 0 (no symptoms present) to 11 (all symptoms present) (1, 20, 23, 24). The secondary endpoints included safety, as indicated by the incidence of treatment-related AEs (defined as any untoward medical occurrence temporally associated with FDC administration, irrespective of its causal relationship to treatment), and evaluation of the influence of demographic subgroups (age and sex), dietary patterns (vegetarian vs nonvegetarian), and comorbidities on treatment response. Efficacy assessments on Days 7 and 12 evaluated improvements in the predefined dyspeptic symptoms present at each scheduled assessment, whereas adverse-event reporting captured events that occurred or were reported during the corresponding follow-up period. New or worsened events were considered AEs when they first occurred after treatment initiation or represented a clinically meaningful worsening of a pre-existing baseline symptom. Baseline symptoms that remained unchanged were not considered AEs.

The other outcome measures included the extent of item-level symptom resolution and patient-level response to the treatment. The item-level symptom resolution was defined, for each of the 11 symptom domains, as a change in status from present at baseline to absent at the follow-up visit, and was expressed as the percentage of patients with that symptom at baseline who did not report it over the subsequent visits. A ≥75% reduction in the composite symptom score from baseline was considered a substantial improvement in overall symptom burden. Patient-level excellent response was defined as the proportion of patients achieving substantial symptom resolution.

#### 2.3.1 Sample size

The sample size was estimated using a one-sample test of proportions, comparing the anticipated treatment-response proportion with a reference response proportion of 49.2%, based on reported response rates in FD trials using the following formula (25, 26):


$$n_{\;=\;\left[Z\alpha /2\surd \left\{p_{0}\left(1-p_{0}\right)\right\}\;+\;Z\beta \surd \{\text{p}1\left(1-\text{p}1\right)\}\right]2^{/}(\text{p}1-p_{0}{)}^{2}}$$

Assuming an anticipated treatment-response proportion of approximately 62% (p_1_ = 0.62), a reference response proportion of 49.2% (p_0_ = 0.492), a two-sided significance level of α = 0.05, and 80% statistical power (1−β), the standard normal deviates were Zα/2 = 1.96 and Zβ = 0.84, respectively. A sample size of 128 patients was estimated to provide 80% statistical power to detect the specified difference between the anticipated and reference response proportions.

To account for an anticipated 20% non-response or attrition rate, the sample size was increased to at least 160 patients, as shown below:


$${\text{n}}_{1}=\;n/\left(1-r\right)$$

where n denotes the calculated sample size (n=128), n__1__ represents the sample size after adjustment for non-response (n_1_=160), and r denotes the anticipated non-response rate (r=0.20). The final sample size of 160 patients was distributed across the four study centers.

### 2.4 Statistical analysis

All enrolled patients with at least one baseline assessment comprised the intention-to-treat (ITT) population, while all patients who completed the Day 12 assessments constituted the per-protocol population. Descriptive statistics were used to summarize demographic characteristics and clinical outcomes. Continuous variables were presented as mean ± standard deviation (SD)/median and interquartile range, while categorical variables were summarized as frequencies and percentages. Normality was assessed using the Shapiro-Wilk/Kolmogorov-Smirnov tests. Continuous variables were analyzed using paired t-tests for symptom scores across visits. Categorical variables were analyzed using the chi-square test (for two groups) or ANOVA (for more than two groups). McNemar’s test was used to evaluate paired categorical data for individual symptom resolution. A *p* value of<0.05 was considered statistically significant. Effectiveness analyses at each visit used observed data from patients assessed at that visit (Baseline: N = 160; Visit 2: n=157; Visit 3: n=144); no imputation was performed for missing symptom-score data arising from loss to follow-up. Data analysis was performed using IBM SPSS (Statistical Package for the Social Sciences) for Windows, version 25.0 (IBM Corp. Armonk, NY; released 2017).

Effectiveness analyses at each follow-up visit used observed data from patients assessed at that visit (complete-case analysis); no imputation was performed for missing symptom-score data arising from loss to follow-up.”

## 3 Results

A total of 160 patients with FD were enrolled and completed baseline assessments. Most patients (n=157; 98.1%) attended the follow-up visit (Visit 2; Day 7), while 144 patients (90.0%) completed the study (Visit 3; Day 12). Overall, 16 patients (10.0%) were lost to follow-up, primarily because they could not be contacted. No withdrawals occurred due to AEs, missed appointments, or protocol violations. The patient disposition flow diagram is presented in **Figure 1**.

**Figure 1:**
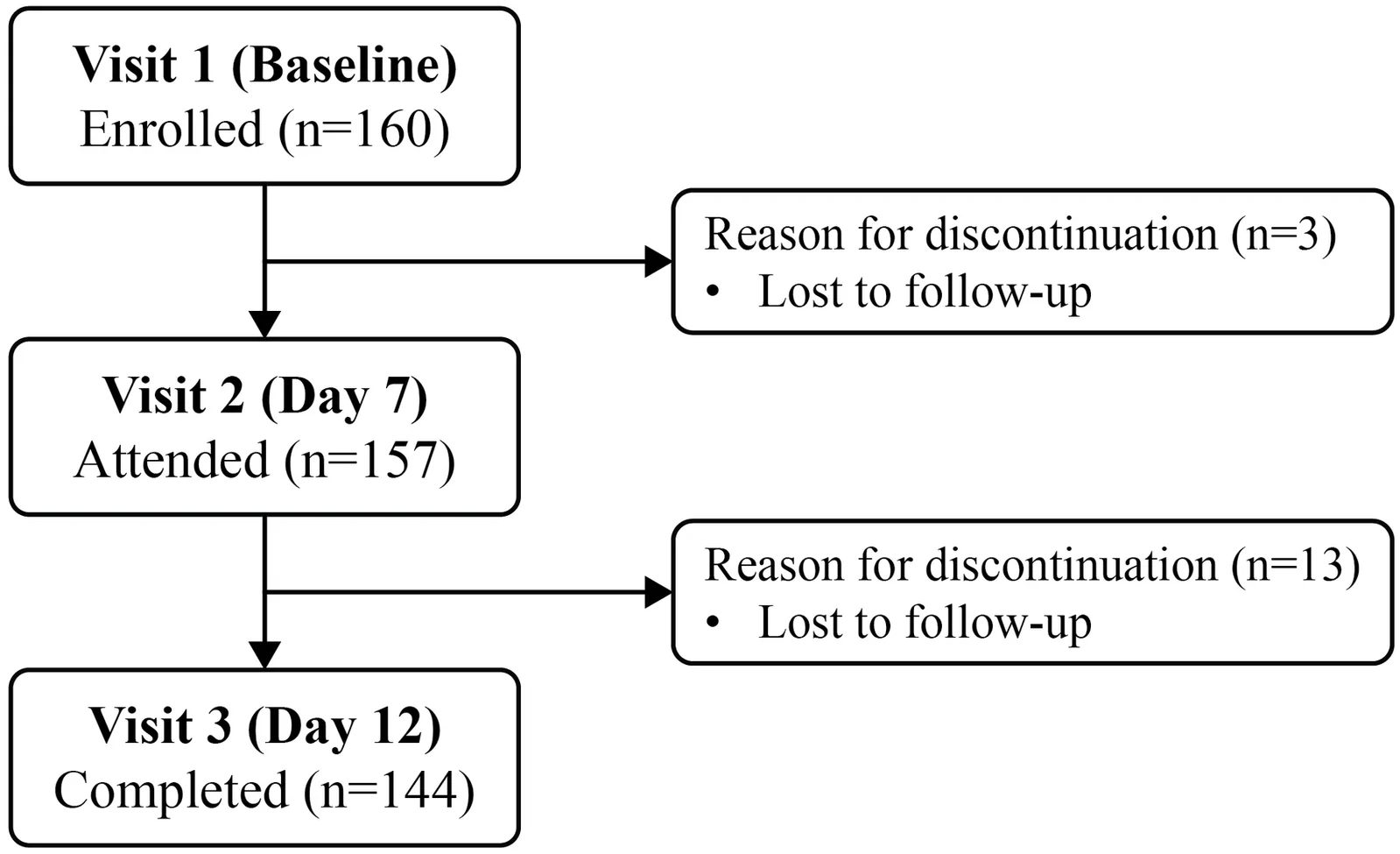
Patient disposition.

### 3.1 Baseline characteristics

Patients had a mean (SD) age of 42.4 ± 13.6 years. Most patients were men (n=85, 53.1%), followed a nonvegetarian diet (n=98, 61.3%), had no comorbidity (n=106, 66.3%), were not taking concomitant medication (n=118, 73.7%), and had not undergone previous treatment for FD (n=94, 58.7%) (**Table 1**).

**Table 1:** Demographic and baseline characteristics of the study population (N = 160).

Characteristic	Value
Age (years; Mean ± SD)	42.4 ± 13.6
Weight (kg; Mean ± SD)	64 ± 6.8
Age category, n (%)	
≤35 years	72 (45.0)
36–55 years	62 (38.8)
>55 years	26 (16.3)
Gender, n (%)	
Male	85 (53.1)
Female	75 (46.9)
Geographic distribution, n (%)	
South	55 (34.4)
North	45 (28.1)
Northeast	40 (25.0)
West	20 (12.5)
Occupation, n (%)	
Housewife	64 (40.0)
Service	46 (28.7)
Business	40 (25.0)
Student	5 (3.1)
Self-employed	3 (1.9)
Retired	2 (1.3)
Medical history, n (%)	
Comorbidities	
Comorbidities present	54 (33.8)
No comorbidities	106 (66.3)
Treatment for FD	
Previous FD treatment	66 (41.3)
No previous FD treatment	94 (58.7)
Concomitant medication	
Concomitant medications	42 (26.3)
No concomitant medications	118 (73.7)
Lifestyle factors, n (%)	
Vegetarian diet	62 (38.8)
Nonvegetarian diet	98 (61.3)

FD, Functional dyspepsia; N, Number of patients at baseline; n: number of patients; SD, Standard deviation; %: Percentage of patients.

Concomitant medications include Atenolol, Amlodipine, Aspirin, Atorvastatin, Clonazepam, Fenofibrate, Glimepiride, Levothyroxine, Losartan, Metformin, Olmesartan, Ondansetron, Pioglitazone, Ramipril, Rosuvastatin, Sitagliptin, Telmisartan, and Zolpidem.

### 3.2 Resolution of symptoms

All patients reported gas problems/flatulence at baseline (n=160, 100%), followed by early satiety/fullness and stomach pain/epigastric pain (n=159, 99.4%, each), heartburn/acid reflux (n=158, 98.8%), and bloating/abdominal distension (n=156, 97.5%). Approximately 49% of patients reported a decline in sleep or overall health quality (n=79; **Supplementary Table S2**). By Visit 2, remarkable symptom resolution was observed (resolution rate, 84.7%–100%), with resolution of nausea/vomiting, regurgitation, burping/belching, stress-related symptoms, constipation, and improvement in sleep/health quality. These improvements were sustained or further improved at Visit 3, with substantial resolution of domain-level metrics (100% each) achieved in all 10 domains except gas problems/flatulence (85.4%; **Supplementary Table S2**). Overall, the unweighted arithmetic mean of domain-specific resolution percentages across the 11 domains as assessed in the study questionnaire was 97.5% (1072.6/11) at Visit 2 and 98.7% (1085.4/11) at Visit 3 (**Figure 2**; **Supplementary Table S2**). By Visit 3, the patient-level excellent response rate was 85.4% (123/144), which reflects the proportion of patients with resolution of all symptoms; gas/flatulence was the residual symptom in the remaining 14.6% (21/144) of patients (**Table 2**).

**Figure 2:**
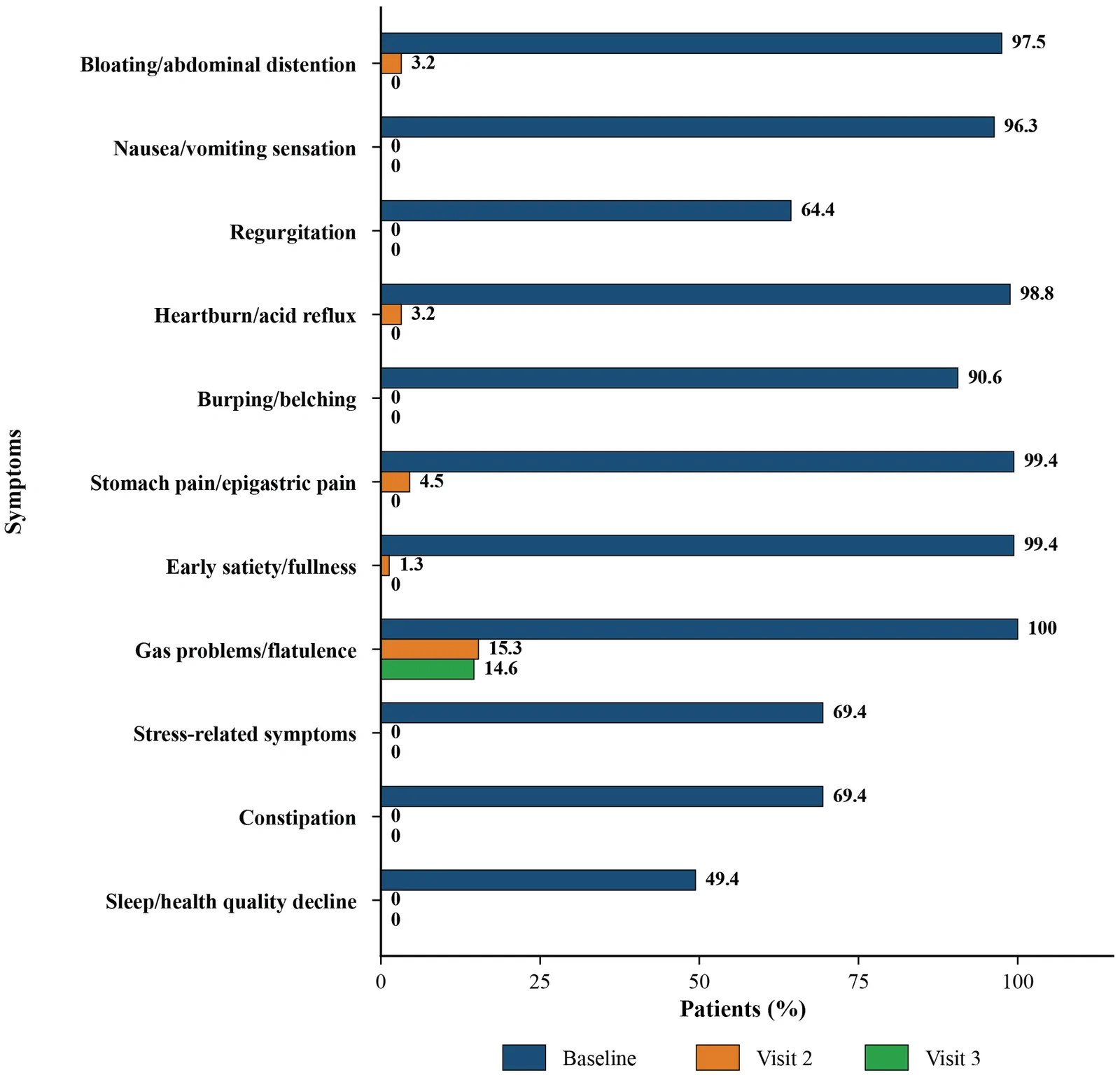
Symptom prevalence and resolution among patients with functional dyspepsia. Number of patients: Baseline, N = 160; Visit 2, n=157; Visit 3, n=144.

**Table 2:** Treatment response analysis across study visits.

Treatment response at Visit 3	No. of patients	No. of responders (n)	Response rate (95% CI)	p value
Excellent response	144	123	85.4% (78.5%–90.7%)	<0.0001*
Residual symptoms	144	21	14.6% (9.3%–21.5%)	<0.0001*

CI, Confidence interval; n, number of patients.

Responders were defined as the patients with a ≥50% reduction in total functional dyspepsia symptom score.

Excellent response (patient-level): ≥75% reduction in mean symptom score.

Statistical test: Binomial exact test comparing observed response rate to a null hypothesis of 49.2%.

*p< 0.05, statistically significant.

### 3.3 Effectiveness of the treatment

#### 3.3.1 Symptom score analysis

Most patients demonstrated a decline in the mean composite symptom score from 9.35 ± 1.42 at baseline to 0.27 ± 0.75 at Visit 2 (Day 7) and 0.15 ± 0.35 at Visit 3 (Day 12) (*p*< 0.0001) (**Figure 3**). The mean improvement from baseline was 97.1% at Visit 2 and 98.4% at Visit 3, with a very large effect size (Cohen’s d=6.5; Day 12).

**Figure 3:**
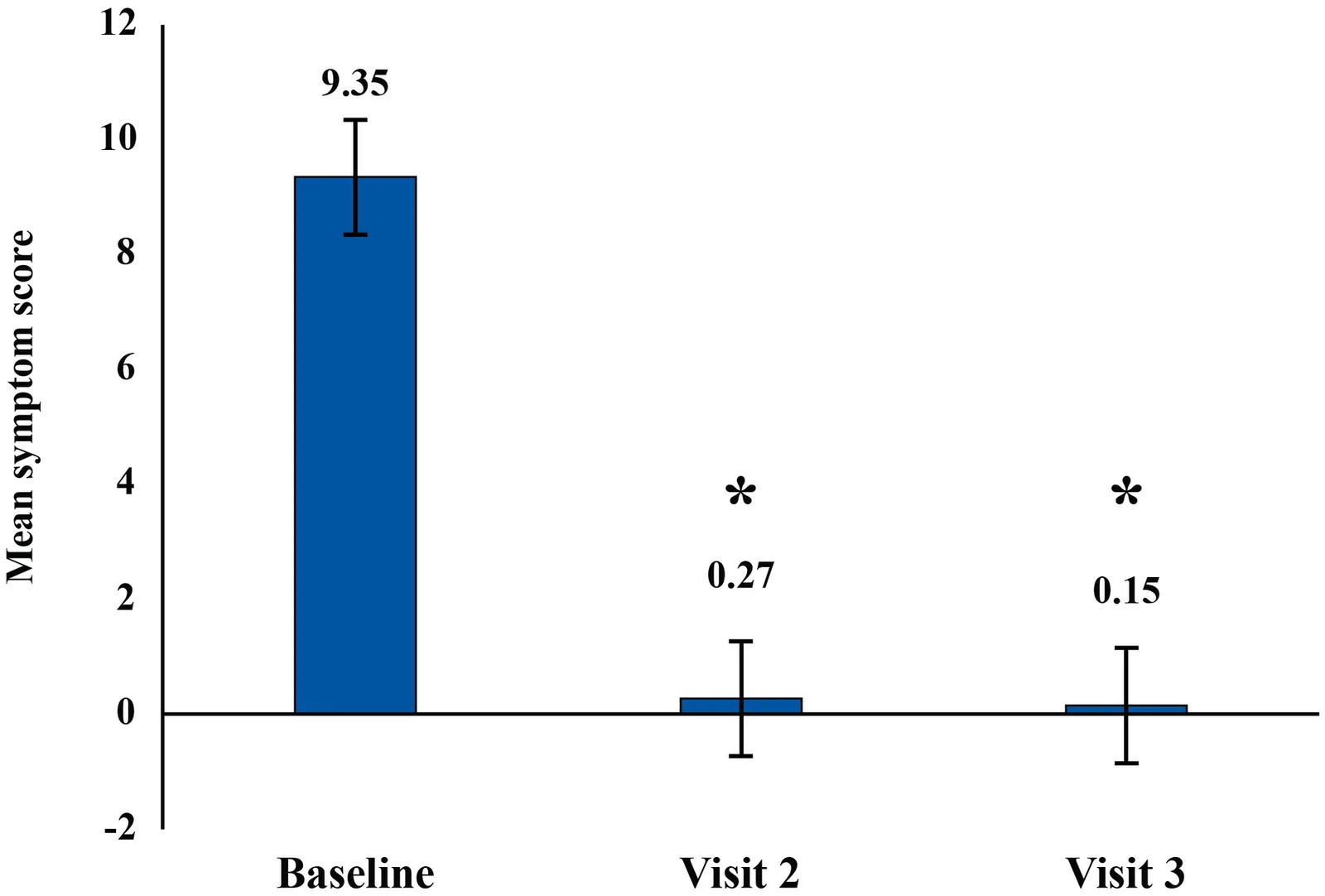
Effect of fixed-dose combination on mean symptom score. The mean symptom score was calculated as the unweighted mean of individual 11-item symptom scores (bloating, nausea/vomiting, regurgitation, heartburn, burping, stomach pain, postprandial fullness/early satiety, gas/flatulence, feeling of stress, constipation, and sleep/overall health quality) using the standardized questionnaire. The responses were recorded dichotomously as present (Yes; score = 1) or absent (No; score = 0) at baseline, Day 7, and Day 12. Paired t-tests were used to compare each follow-up visit with baseline. *p< 0.0001.

The mean time to first symptom relief was 4.11 ± 0.41 hours (n=151), while maximum clinical benefit was observed in 141 patients, with a mean time of 3.80 ± 1.23 days. All patients experienced initial symptom relief during the onset phase (the period following the first FDC administration during which the patient experiences symptom relief; 0–6 hours) and achieved stabilization of symptom control or clinical response during the consolidation phase (a short-term management stage aimed at achieving symptom remission; 1–7 days). By the maintenance phase (a long-term management strategy following successful initial treatment; 7–12 days), 85.4% of patients demonstrated full symptom resolution (**Supplementary Table S3**).

#### 3.3.2 Treatment response analysis

On Day 7, all patients assessed at Visit 2 demonstrated a clinical response, yielding a 100% response rate (157/157; 95% CI, 97.7–100.0; *p*< 0.0001), which was maintained at Day 12 (Visit 3; 144/144; 95% CI, 97.5–100.0; *p*< 0.0001). Overall, 85.4% (123/144) of patients achieved a excellent response (95% CI, 78.5%–90.7%; *p*< 0.0001) at Day 12, while 14.6% (21/144) exhibited residual symptoms (**Table 2**).

### 3.4 Safety of the intervention

Overall, 20 patients reported one or more AEs, including bloating, nausea, vomiting, stomach upset, and fever, during the study. The incidence of AEs was lower on Day 12 (n=6, 4.2%) than on Day 7 (n=14, 8.9%). No serious adverse events were reported, including death, life-threatening events, hospitalization or prolonged hospitalization, persistent or significant disability/incapacity, congenital anomalies/birth defects, or other medically important events. High protocol adherence was observed, with 98.1% (157/160) and 90.0% (144/160) of patients completing Visits 2 and 3 per protocol, respectively. Discontinuations were primarily due to loss to follow-up rather than safety concerns (**Table 3**).

**Table 3:** Safety analysis.

Safety parameter	Visit 2 (n=157)	Visit 3 (n=144)	Overall (N = 160)	95% CI
AEs				
Any AE	14 (8.9%)	6 (4.2%)	20 (12.5%)	8.2%–18.5%
Study completion				
Completed per protocol	157 (98.1%)	144 (90.0%)	144 (90.0%)	84.4%–93.7%
Lost to follow-up	3 (1.9%)	13 (8.1%)	16 (10.0%)	6.3%–15.6%

AE, Adverse event; CI, Confidence interval; n, Number of patients; N, Number of patients at baseline.

The confidence intervals were calculated using the Wilson score method with the correct overall denominator of 160.

At Visit 2, AEs included excessive gas, bloating, nausea, vomiting, stomach upset, fever, abdominal cramps, weakness, and heartburn.

At Visit 3, AEs included bloating, nausea, vomiting, stomach upset, and fever.

The patients with FD who reported adverse events at Visit 2 (n=14) and Visit 3 (n=6) were distinct individuals, with no overlap between them.

#### 3.4.1 Subgroup analysis

Subgroup analysis demonstrated significant improvements in mean symptom scores from baseline to Visit 2 among patients aged 36–55 years (*p*< 0.01) and those with comorbidities (*p* = 0.01). However, no significant differences in treatment response were observed across gender, age groups (≤35 years and >55 years), dietary habits, previous treatment history, or geographic regions at Visit 2 (*p >*0.05) (**Supplementary Tables S4**, **S5**).

## 4 Discussion

This prospective, multicenter, real-world, single-arm, interventional study demonstrated the short-term effectiveness of a FDC of papain and alpha-amylase in managing FD over a 12-day treatment period, with significant, rapid, and consistent symptom improvement and a favorable safety profile. Administration of the FDC resulted in a statistically significant and early reduction in overall FD symptom burden, as reflected by more than 97% improvement in mean composite symptom scores from baseline by the first follow-up visit (Day 7; p<0.001), which was sustained through study completion (Day 12; *p*< 0.0001). The nominal mean score on Day 12 (0.15 ± 0.35) reflects the residual item-level symptom count due to gas/flatulence. All patients assessed for symptom relief experienced initial relief within 4 hours of starting therapy, and 85.4% achieved an excellent response within 7–12 days. Improvements were observed across symptoms, including bloating, nausea, regurgitation, heartburn, early satiety, and epigastric pain.

These short-term findings are broadly consistent with an earlier randomized, placebo-controlled study, which reported that a multienzyme complex consisting of five digestive enzymes: alpha-amylase, protease, lipase, lactase, and cellulase, was effective and safe in alleviating dyspeptic symptoms (14). Similarly, another randomized clinical trial found that multidigestive enzyme blends containing protease, amylase, lipase, cellulase, and lactase reduced FD symptoms and improved sleep quality among patients (19). However, these comparator trials differed from the present study in design (randomized, placebo- or active-controlled vs. single-arm, real-world), enzyme composition, and follow-up duration. Therefore, direct comparisons of the magnitude of treatment effects should be interpreted with caution.

The observed clinical benefits of this FDC over the 12-day period may be attributed to its complementary enzymatic actions. Papain is known to enhance proteolysis and reduce postprandial gastric workload, while alpha-amylase (fungal diastase) facilitates carbohydrate digestion, while limiting excessive fermentation (16–18, 27). The underlying mechanism of action is multifactorial, wherein through improved digestive efficiency, this enzyme combination may alleviate intragastric distension, reduce upper abdominal discomfort, and improve postprandial fullness, all of which are hallmark features of FD. However, lipase was not included in the FDC because fat maldigestion or pancreatic exocrine insufficiency was not a predefined eligibility criterion or therapeutic target. In addition, prior evidence suggests that enzymatic supplementation may indirectly modulate gut motility and attenuate postprandial inflammatory responses by optimizing luminal contents, further contributing to symptom relief (15, 19). The magnitude of short-term symptom relief observed with the FDC compares favorably with outcomes for conventional pharmacological therapies for FD, although such comparisons are indirect given differences in study design, patient populations, and follow-up duration. Further comparative studies are needed also to determine whether the addition of lipase provides any incremental clinical benefit. Earlier studies evaluating PPIs and prokinetic agents have typically reported satisfactory symptom relief after 4–8 weeks of treatment. For example, DA-9701 (Motilitone), alone or in combination with pantoprazole over 4 weeks, improved global symptoms in 60.5% and 63.5% of patients with FD, respectively (28). The SAMURAI study reported that rabeprazole (20 mg once daily) provided satisfactory symptom relief in 45.3% of patients with FD (vs placebo, 28.2%), based on the dyspepsia symptom questionnaire over a multi-week follow-up (29). However, acid-modulating agents, including PPIs, H2RAs, as well as prokinetics and neuromodulatory agents such as buspirone and mirtazapine, require prolonged treatment durations to achieve optimal efficacy in functional gastrointestinal disorders, including FD (12, 28–30).

In the present study, the papain and alpha-amylase combination demonstrated symptom improvement within the 12-day treatment period, with a favorable short-term tolerability profile characterized predominantly by mild treatment-related AEs in a minority of patients. High treatment adherence was observed, as reflected by a 90.0% study completion rate, with no requirement for dose modifications. These attributes are particularly relevant in FD, a chronic and relapsing disorder in which long-term adherence is frequently compromised by adverse effects associated with acid-suppressive, prokinetic, or centrally acting agents (12, 15). In contrast, long-term use of PPIs and H2RAs has been associated with potential safety concerns, including renal complications, infections, and micronutrient deficiencies (31–33). A case-control study reported that chronic use (≥12 months) of H2RAs or PPIs was associated with a significantly increased risk of vitamin B12 deficiency (OR, 4.45; 95% CI, 1.47–13.34) (34). Similarly, another nested case-control study documented an elevated risk of vitamin B12 deficiency with exposure to PPIs or H2RAs for ≥2 years (OR, 1.65; 95% CI, 1.58–1.73 and OR, 1.25; 95% CI, 1.17–1.34, respectively) (35). Prokinetic agents may also have specific safety considerations; for example, cisapride has been associated with dose-dependent cardiac adverse effects, including QT interval prolongation, syncope, and ventricular arrhythmias (36). Another randomized clinical trial reported AEs (such as nausea, diarrhea, and vomiting) in 17.8%, 16.8%, and 11.9% of patients with FD treated with DA-9701 (Motilitone) or pantoprazole, either alone or in combination, respectively (28). However, as these safety concerns were generally evaluated with prolonged exposure, differences in study design, treatment duration, and interventions should be considered when interpreting them as contextual rather than direct comparisons with the present 12-day study.

The strength of this study lies in its real-world, multicenter design, which enhances external validity and captures outcomes reflective of everyday clinical practice. The use of validated Rome IV criteria adds methodological rigor. Despite these strengths, the findings should be interpreted with consideration of certain limitations. The study employed a single-arm design without a placebo or comparator group, precluding definitive causal inference. The short 12-day duration and the absence of post-treatment follow-up limit conclusions strictly to the short-term symptomatic response observed during active treatment; no inference can be drawn regarding relapse, durability of benefit, or long-term safety after treatment discontinuation. In addition, concomitant medication effects, formal adverse-event causality assessment, and treatment adherence were not systematically evaluated, as these were beyond the prespecified scope of the study; therefore, their potential influence on the observed efficacy and safety outcomes cannot be determined. Although symptom relief was consistent, self-reported outcomes may introduce bias. Nevertheless, the consistent response observed across subgroups makes random variability an improbable determinant of the observed outcomes.

Concomitant medication effects, formal adverse-event causality assessment, and treatment adherence were not systematically evaluated, as these were beyond the prespecified scope of the study. Therefore, their potential influence on the observed efficacy and safety outcomes cannot be excluded.

In conclusion, the present real-world, single-arm interventional study provides preliminary evidence that a FDC of papain and alpha-amylase offers rapid, clinically meaningful symptom relief in FD over a 12-day treatment period, with favorable short-term safety, tolerability, and treatment adherence. These findings support the potential utility of digestive enzyme supplementation as a therapeutic option for FD, particularly for individuals who experience suboptimal benefit or intolerance to conventional therapies. However, given the single-arm design and short treatment duration, the findings do not establish long-term efficacy, durability of response, or safety with extended use. Evaluation of biochemical markers of digestion and gastric motility may also provide additional insights into the therapeutic mechanisms of enzymatic supplementation in FD. Future longer-term, randomized controlled studies are warranted to confirm these findings, evaluate the durability of response and safety over extended treatment periods, and establish the position of enzyme-based therapy within FD treatment algorithms.

## Data Availability

The original contributions presented in the study are included in the article/**Supplementary Material**. Further inquiries can be directed to the corresponding author.
