# Supplementary material for: Efficacy and safety of a fixed-dose papain and alpha-amylase combination in patients with functional dyspepsia: a real-world study
Source: Front Gastroenterol (Lausanne). 2026 Oct 28;5:1954358. doi: 10.3389/fgstr.2026.1954358 (PMC13751347; doi:10.3389/fgstr.2026.1954358)
Supplement: Supplementary file 1 [file Table1.docx]

**Efficacy and Safety of a Fixed-Dose Papain and Alpha-Amylase Combination in Patients with Functional Dyspepsia: A Real-World Study**

**Supplementary information**

**Supplementary Table S1. Study questionnaire**

| **No.** | **Question** | **Response** |
| --- | --- | --- |
| 1 | Do you experience your belly feeling full and tight (bloating)? | Yes/No |
| 2 | Do you experience feelings of vomiting (nausea)? | Yes/No |
| 3 | Do you experience when swallowed food comes back up (regurgitation)? | Yes/No |
| 4 | Do you experience heartburn? | Yes/No |
| 5 | Do you experience burping? | Yes/No |
| 6 | Do you experience stomach pain? | Yes/No |
| 7 | Do you experience a feeling of fullness after eating very little? | Yes/No |
| 8 | Do you have a problem with gas in your stomach? | Yes/No |
| 9 | Do you feel stressed? | Yes/No |
| 10 | Do you have constipation issues? | Yes/No |
| 11 | Have you observed a decline in your health, including weakness or quality of sleep? | Yes/No |

Each of the 11 symptoms displayed in the questionnaire was recorded dichotomously as present (Yes; score = 1) or absent (No; score = 0).

**Supplementary Table S2. Symptom prevalence and resolution analysis**

| **Symptom domain** | **Baseline, N (%)** | **Visit 2, n (%)** | **Visit 3, n (%)** | **Resolution rate at Visit 2 (%)** | **Resolution rate at Visit 3 (%)** | **McNemar p-value** |
| --- | --- | --- | --- | --- | --- | --- |
| Bloating/abdominal distension | 156 (97.5) | 5 (3.2) | 0 (0.0) | 96.8 | 100.0 | <0.0001 |
| Nausea/vomiting sensation | 154 (96.3) | 0 (0.0) | 0 (0.0) | 100.0 | 100.0 | <0.0001 |
| Regurgitation | 103 (64.4) | 0 (0.0) | 0 (0.0) | 100.0 | 100.0 | <0.0001 |
| Heartburn/acid reflux | 158 (98.8) | 5 (3.2) | 0 (0.0) | 96.8 | 100.0 | <0.0001 |
| Burping/Belching | 145 (90.6) | 0 (0.0) | 0 (0.0) | 100.0 | 100.0 | <0.0001 |
| Stomach pain/epigastric pain | 159 (99.4) | 7 (4.5) | 0 (0.0) | 95.6 | 100.0 | <0.0001 |
| Early satiety/fullness | 159 (99.4) | 2 (1.3) | 0 (0.0) | 98.7 | 100.0 | <0.0001 |
| Gas problems/flatulence | 160 (100.0) | 24 (15.3) | 21 (14.6) | 84.7 | 85.4 | <0.0001 |
| Stress-related symptoms | 111 (69.4) | 0 (0.0) | 0 (0.0) | 100.0 | 100.0 | <0.0001 |
| Constipation | 111 (69.4) | 0 (0.0) | 0 (0.0) | 100.0 | 100.0 | <0.0001 |
| Sleep/health quality decline | 79 (49.4) | 0 (0.0) | 0 (0.0) | 100.0 | 100.0 | <0.0001 |
| **Arithmetic mean (unweighted) of domain-specific resolution percentages** |  |  |  | **97.5** | **98.7** | **<0.0001** |

Statistical test: McNemar's test, follow-up visits vs. baseline; p<0.05, statistically significant.

Number of patients: Baseline, N=160; Visit 2, n=157; Visit 3, n=144
Resolution rate: Percentage of patients with baseline symptoms who achieved symptom resolution

Arithmetic mean (unweighted) of domain-specific resolution percentages = [Resolution rate X number of domains (symptoms)]/11

**Supplementary Table S3. Time to symptom relief and treatment kinetics**

| **Time parameter** | **n** | **Mean ± SD** |
| --- | --- | --- |
| Time to first symptom relief (hours)† | 151 | 4.11 ± 0.41 |
| Time to maximum benefit (days)‡ | 141 | 3.80 ± 1.23 |
| **Treatment kinetics analysis** | | |
| **Phase** | **n** | **% Patients^#^** |
| Onset phase (0–6 hours) | 151 | 100.0% |
| Consolidation phase (1–7 days) | 157 | 100.0% |
| Maintenance phase (7–12 days) | 144 | 85.4% |

n: Number of patients; SD: Standard deviation.

†Assessed at Visit 2 (day 7); 6 patients did not record or report relief values, and 3 were lost to follow-up before Visit 2 (day 7)

‡Assessed at Visit 3 (day 12); 3 patients did not record relief values in days, and 13 patients were lost to follow-up before Visit 3 (day 12)

**^#^** Percentage of patients with symptom resolution

**Supplementary Table S4. Subgroup analysis**

| **Subgroup** | **No. of patients at Visit 2** | **Mean symptom score (Baseline)** | **Mean symptom score (Visit 2)** | **Mean reduction (Visit 2)** | **p-value†** |
| --- | --- | --- | --- | --- | --- |
| **Gender** | | | | | |
| Male | 84 | 9.88 ± 1.33 | 0.35 ± 0.96 | 9.54 ± 1.49 | 0.21 |
| Female | 73 | 8.74 ± 1.31 | 0.19 ± 0.40 | 8.55 ± 1.27 | — |
| **Age** |  |  |  |  |  |
| ≤35 years | 71 | 9.24 ± 1.47 | 0.51 ± 1.04 | 8.73 ± 1.55 | — |
| 36–55 years | 61 | 9.64 ± 1.45 | 0.07 ± 0.25 | 9.57 ± 1.41 | < 0.01 |
| >55 years | 25 | 8.96 ± 1.21 | 0.12 ± 0.33 | 8.84 ± 1.07 | 0.07 |
| **Diet** | | | | | |
| Vegetarian | 59 | 9.07 ± 1.22 | 0.17 ± 0.38 | 8.90 ± 1.17 | 0.18 |
| Nonvegetarian | 98 | 9.52 ± 1.53 | 0.34 ± 0.91 | 9.18 ± 1.63 | — |
| **Comorbidities** |  |  |  |  |  |
| No comorbidities | 107 | 9.31 ± 1.52 | 0.37 ± 0.89 | 8.93 ± 1.57 | — |
| With comorbidities | 50 | 9.44 ± 1.25 | 0.06 ± 0.24 | 9.38 ± 1.19 | 0.01 |
| **Previous treatment** | | | | | |
| Treatment-naïve | 92 | 8.99 ± 1.39 | 0.36 ± 0.93 | 8.63 ± 1.44 | — |
| Previously treated | 65 | 9.86 ± 1.36 | 0.15 ± 0.36 | 9.71 ± 1.28 | 0.09 |

Statistical test: Independent t-test (continuous variables); Chi-square test (categorical variables); †p<0.05, statistically significant.

**Supplementary Table S5. Geographic subgroup analysis**

| **Geographical region** | **No. of patients enrolled (Baseline, N)** | **Mean symptom score (Baseline)** | **Mean symptom score (Visit 2)** | **Mean reduction (Visit 2)** | **p-value†** |
| --- | --- | --- | --- | --- | --- |
| South | 55 | 9.33 ± 1.35 | 0.09 ± 0.29 | 9.24 ± 1.38 | 0.054 |
| North | 45 | 9.51 ± 1.22 | 0.23 ± 0.43 | 9.30 ± 1.29 | — |
| West | 20 | 9.50 ± 1.28 | 0.26 ± 0.45 | 9.26 ± 2.22 | 0.799 |
| Northeast | 40 | 9.10 ± 1.78 | 0.57 ± 1.32 | 8.53 ± 1.38 | 0.110 |

SD, Standard Deviation; V1, visit 1 or baseline; V2, Visit 2; V3, Visit 3

Number of patients: Baseline: 160; V2: 157; V3: 144.

Statistical test: ANOVA; *p<0.05, statistically significant.
